# Prognostic impact of nutritional indicators based on Lasso-Cox regression for non-muscle-invasive bladder cancer

**DOI:** 10.3389/fnut.2025.1560655

**Published:** 2025-04-28

**Authors:** Junjiang Ye, Yandong Xie, Biao Ran, Ping Han

**Affiliations:** Department of Urology, Institute of Urology, West China Hospital, Sichuan University, Chengdu, China

**Keywords:** non-muscle-invasive bladder cancer, Lasso-Cox regression, geriatric nutritional risk index, prognostic nutritional index, Naples prognostic score

## Abstract

**Background:**

There is a lack of prognostic models to predict the outcomes of non-muscle-invasive bladder cancer (NMIBC) patients receiving Bacillus Calmette-Guérin (BCG) immunotherapy. Existing nutritional risk indicators, such as the prognostic nutritional index (PNI), geriatric nutritional risk index (GNRI) and Naples prognostic score (NPS), have demonstrated prognostic value in various malignancies. This study aimed to construct novel nutritional risk indexes (NRIs) using peripheral blood markers via Lasso-Cox regression and validate their prognostic value.

**Methods:**

The electric medical records in our institution were searched and data of 525 NMIBC patients were collected. The Lasso-Cox regression was employed to screen preoperative blood biomarkers correlated with recurrence-free survival (RFS), time to BCG-treatment failure (TTF), and progression-free survival (PFS). NRIs were developed based on selected markers and validated against GNRI, PNI, NPS, and the EAU2021 risk model using Kaplan–Meier analysis, Cox regression, receiver-operating characteristic (ROC) curves, Concordance index (C-index) and Decision Curve analysis.

**Results:**

Lasso-Cox regression identified distinct blood biomarkers: gamma-glutamyl transpeptidase (GGT), serum total protein (TP), albumin and cholesterol were predictive of tumor recurrence and BCG failure, while GGT, TP, and coefficient variation of red blood cell volume distribution width were linked to tumor progression. Three NRIs—NRITR (RFS), NRIBF (TTF) and NRITP (PFS)—were constructed. The NRIs exhibited prognostic value through Kaplan–Meier analysis. Multivariate Cox analysis confirmed NRITR (HR = 0.38, 95%CI:0.28–0.53), NRIBF (HR = 0.45, 95%CI: 0.30–0.67), and NRITP (HR = 0.38, 95%CI: 0.21–0.69) as independent predictors. Nomograms incorporating NRIs demonstrated superior discriminative performance in predicting RFS (AUC = 0.739, C-index = 0.673), TTF (AUC = 0.795, C-index = 0.767), and PFS (AUC = 0.796, C-index = 0.788), and could bring more net benefit for NMIBC patients.

**Conclusion:**

The Lasso-Cox regression may offer superior value in selecting prognostic biomarkers for NMIBC. The Lasso-Cox regression based NRIs enhance prognostic stratification for BCG-treated NMIBC, outperforming existing blood-based nutritional risk indicators and the EAU2021 model. Incorporation of blood-based nutritional indicators into clinical practice could optimization of personalized NMIBC treatment strategies and clinical decision-making. Further validation is warranted.

## 1 Introduction

Bladder cancer (BCa) is recognized as the 9th most prevalent carcinoma worldwide, accounting for 220,000 deaths in 2022 (1). Non-muscle-invasive bladder cancer (NMIBC), which constitutes approximately 75% of all BCa cases, is characterized by high rates of recurrence and progression (2). Transurethral resection of bladder tumor (TURBT) represents the gold standard treatment for NMIBC, and Bacille Calmette-Guérin (BCG) has demonstrated significant effectiveness as an adjuvant intravesical treatment (3). Nonetheless, 20%–40% of patients receiving BCG experience treatment failure, heightening the likelihood of an unfavorable prognosis (4). For BCG failure cases, the primary treatment options include radical cystectomy (RC) or bladder-preserving strategies (e.g., intravesical chemotherapy, device-assisted therapies, or systemic immunotherapy). Due to the significant morbidity associated with RC, bladder-preserving strategies are increasingly preferred (3). Current prognostic tools for NMIBC—such as the 2021 European Association of Urology risk stratification model (EAU2021), European Organisation for Research and Treatment of Cancer scoring model (EORTC), and Club Urologico Español de Tratamiento Oncologico scoring model—are widely used but exhibit limited accuracy in BCG-treated populations (5, 6). This limitation stems from the absence of standardized TURBT and BCG cohorts during their development. Current studies showed that blood-based nutritional indicators exhibit robust prognostic predictive ability in cancer outcomes. These biomarkers could thus serve as a practical complement to existing NMIBC prognostic models, facilitating more tailored treatment strategies based on individual patient profiles.

Malignancy, as a metabolically demanding disease, is distinctly characterized by malnutrition, with approximately one-third of cancer patients exhibiting heightened nutritional risk (5). Notably, these nutritional challenges are evident even in the early stages of the disease (7). Tailoring malnutrition assessment to the specific postoperative risks of each cancer type can enhance personalized care and potentially improve disease outcomes (5). Significantly, as a modifiable disease state, early detection of malnutrition is critical for improving quality of life, reducing economic burdens, and optimizing therapeutic responses. However, the complexity in identifying malnutrition may lead to treatment delays and potentially exacerbate conditions among cancer patients (8). Existing validated nutritional assessment tools, such as the Nutritional Risk Screening 2002, the Malnutrition Universal Screening Tool, and the Mini Nutritional Assessment (MNA), have showed limited operability in clinical practice (9). And research has predominantly focused on the role of malnutrition in progressive or late-stage malignancies. Blood serves as a key medium for assessing nutritional status. Peripheral blood biomarkers are cost-effective, readily accessible, and highly sensitive, making them widely used for clinical malnutrition evaluation and prognosis prediction. A meta-analysis of 111 studies (*n* = 52,911) demonstrated significantly lower levels of serum albumin, total cholesterol, total protein and hemoglobin in high-risk malnutrition groups compared to controls, highlighting the potential of hematologic nutritional indicators in reflecting nutritional status. The Naples prognostic score (NPS) has demonstrated independent prognostic value in upper tract urothelial carcinoma (10). In BCa, indicators like the prognostic nutritional index (PNI) and Geriatric Nutritional Risk Index (GNRI) have shown associations with patient prognosis in several studies, though evidence remains limited (11, 12).

Given the limited research on the association between nutritional indicators and NMIBC outcomes, we innovatively applied the Lasso-Cox regression model to select and construct nutritional risk indicators (NRIs) based on peripheral blood-based nutritional Indices, and evaluated the prognostic value of the nutritional Indicators in NMIBC patients undergoing intravesical BCG therapy.

## 2 Materials and methods

### 2.1 Date collection and selection

Between September 2018 and June 2024, patients with NMIBC undergoing BCG treatment at our institution was screened, following approval from the institutional ethics committee (No.2023128). Key inclusion criteria were: (1) patients pathologically diagnosed with NMIBC after TURBT at our institution and receiving standard BCG intravesical therapy; (2) availability of blood data obtained 2-week before surgery; (3) routine follow-up through outpatient and telephone for more than 12 months. Key exclusion criteria included: (1) receipt of other intravesical chemotherapies, with the exception of immediate single post-TURBT instillations; (2) presence of concurrent malignancies or other conditions potentially impacting blood-biochemical and hematological measurements; (3) incomplete data acquisition.

Demographic, clinicopathological characteristics, and preoperative serum laboratory data were extracted from the electronic health records of the enrolled patients. T stages and carcinoma *in situ* (CIS) were classified according to the 2017 American Joint Committee on Cancer TNM staging system (8th edition). Tumor grading was conducted in accordance with the 2004/2016 WHO grading system. The PNI was defined as total lymphocyte count (10^9^/L) × 5 + serum albumin (g/L). GNRI was defined as 1.489 × serum albumin (g/L) + 41.7 × body mass (kg)/ideal body mass (kg), with the ideal body mass calculated using the Lorentz Formula. NPS was derived from the aggregate scores, categorized into three tiers as illustrated in Supplementary Figure 1.

### 2.2 Patient management and follow-up

The BCG treatment regimen commenced with an induction dose of 120 mg, administered weekly for six consecutive weeks. Patients then entered a maintenance phase with bi-weekly instillations for at least 1 year. Follow-up included cystoscopy and urinary cytology every 3 months for 2 years, semi-annually in the third year, and annually thereafter.

During follow-up, tumor recurrence and progression were verified pathologically. Recurrence-free survival (RFS) was measured from the initial TURBT to documented recurrence. Progression-free survival (PFS) was the time from surgery to muscle-invasive disease or metastasis. EAU guidelines define BCG failure as high-grade disease emergence during or after BCG therapy, with time to BCG-treatment failure (TTF) being from surgery to muscle-invasive disease or local high-grade recurrence. Unresponsive tumors encompass refractory tumors and those exhibiting T1/Ta high-grade recurrence within 6 months or CIS within 12 months following adequate BCG exposure. Late BCG relapse is defined as Ta/T1 high-grade recurrence or CIS beyond 6 or 12 months of last BCG exposure (3).

### 2.3 Statistical analysis

Descriptive and analytical methods processed data. All candidate peripheral blood nutritional indicators—including red blood cell count, white blood cell count, hemoglobin, platelet count, red blood cell distribution (RDW), neutrophils count, eosinophils count, lymphocyte count, basophils count, monocyte count, fibrinogen, alanine aminotransferase, aspartate aminotransferase, alkaline phosphatase, gamma-glutamyl transpeptidase (GGT), lactate dehydrogenase, hydroxybutyrate dehydrogenase, serum total protein (TP), serum albumin, serum globulin, serum albumin-to-globulin ratio, low-density lipoprotein, high-density lipoprotein, triglycerides, cholesterol, and total bilirubin—were incorporated into an L1-penalized Cox regression framework. Model complexity parameters (penalty coefficient *λ*) were optimized via 10-fold cross-validation for feature selection, followed by construction of a novel nutritional prognostic index based on non-zero coefficient indicators corresponding to the optimal *λ* value (Lasso-Cox regression). Referring to the results of the Lasso-Cox regression (Supplementary Figure 2), NRI for tumor recurrence (NRITR) consisted of GGT, TP, albumin and cholesterol. The formula was: NRITR = GGT ∗ (0.0023) + TP ∗ (−0.0192) + albumin ∗ (−0.0456) + cholesterol ∗ (−0.0039). Similarly, the formula of NRI for BCG-treatment failure (NRIBF) was: NRIBF = GGT ∗ (0.0040) + TP ∗ (−0.0287) + albumin ∗ (−0.0145*) + cholesterol ∗ (−0.0028). And the formula of NRI for tumor progression (NRITP) was: NRITP = GGT ∗ (0.0014) + TP ∗ (−0.0456) + RDW ∗ (0.0008).

Categorical and continuous variables were compared across NRITR, NRIBF, NRITP, and EAU risk groups using Chi-squared and Kruskal-Wallis tests. Optimal nutritional indicator cut-offs were identified using the receiver operating characteristic (ROC) curves and the Youden index. Prognostic analyses for RFS, TTF, and PFS were performed with validation by Kaplan–Meier and log-rank tests, followed by Cox regression to evaluate the link between nutrition and prognosis. Considering the potential multicollinearity among the nutrition indicators, only one nutrition-based indicator was added in the multivariate Cox proportional hazards regression analysis. Then, according to the results of Cox regression, predictive nomograms incorporating nutritional risk indicators were constructed. The concordance index (C-index), ROC analysis and Decision Curve analysis determined the prognostic value of the nutritional indicator-based prognostic models. Statistical significance was set at the *p* < 0.05 threshold. All analyses were conducted using R software, version 4.4.0.

## 3 Results

### 3.1 Demographic and clinicopathological characteristics of the patients

The study cohort comprised 525 patients (Supplementary Figure 3), with a median follow-up period of 38.1 months. As detailed in Table 1, the median patient age was 67 years. 261 patients were categorized in the high or very-high risk by EAU NMIBC risk stratification. During BCG treatment, 176 (33.5%) patients had tumor recurrence and 54 (10.3%) had progression. Of these, 416 (79.2%) responded to BCG, 84 (16.0%) had late relapses, and 25 (4.8%) were non-responders. The EAU2021 model categorized most clinical characteristics (Supplementary Table 1).

**Table 1:** Demographic and clinical data of patients with NMIBC stratified by NRITR, NRIBF, and NRITP.

Characteristics	Total	NRITR		*p* Value	NRIBF		*p* Value	NRITP		*p* Value
		High	Low		High	Low		High	Low	
Number of patients	525	106	413		191	328		245	274	
Tumor recurrence, *n*(%)				**<0.001**			**<0.001**			**<0.001**
No	349 (66.48)	42 (39.62)	303 (73.37)		96 (50.26)	250 (76.22)		134 (54.69)	212 (77.37)	
Yes	176 (33.52)	64 (60.38)	110 (26.63)		95 (49.74)	78 (23.78)		111 (45.31)	62 (22.63)	
Tumor progression, *n*(%)				**0.001**			**<0.001**			**<0.001**
No	471 (89.71)	85 (80.19)	381 (92.25)		157 (82.20)	309 (94.21)		207 (84.49)	259 (94.53)	
Yes	54 (10.29)	21 (19.81)	32 (7.75)		34 (17.80)	19 (5.79)		38 (15.51)	15 (5.47)	
BCG response				**<0.001**			**<0.001**			**<0.001**
Responsive	416 (79.24)	66 (62.26)	345 (83.54)		126 (65.97)	285 (86.89)		171 (69.80)	240 (87.59)	
Unresponsive	25 (4.76)	9 (8.49)	16 (3.87)		14 (7.33)	11 (3.35)		14 (5.71)	11 (4.01)	
Late relapsing	84 (16.00)	31 (29.25)	52 (12.59)		51 (26.70)	32 (9.76)		60 (24.49)	23 (8.39)	
EAU2021 risk groups, *n*(%)				0.090			**0.018**			**0.002**
Low	99 (18.86)	10 (9.43)	87 (21.07)		22 (11.52)	75 (22.87)		28 (11.43)	69 (25.18)	
Intermediate	165 (31.43)	34 (32.08)	129 (31.23)		59 (30.89)	104 (31.71)		79 (32.24)	84 (30.66)	
High	224 (42.67)	53 (50.00)	169 (40.92)		96 (50.26)	126 (38.41)		119 (48.57)	103 (37.59)	
Very high	37 (7.05)	9 (8.49)	28 (6.78)		14 (7.33)	23 (7.01)		19 (7.76)	18 (6.57)	
Age, M(Q₁,Q₃)	67.00 (57.00,74.00)	71.00 (65.00,80.00)	66.00 (56.00,73.00)	**<0.001**	68.00 (58.00,77.00)	66.00 (56.00,73.00)	0.106	68.00 (60.00,77.00)	65.50 (55.00,72.00)	**<0.001**
BMI, M(Q₁,Q₃)	23.66 (21.63,25.80)	23.24 (21.10,25.53)	23.78 (21.72,25.95)	0.293	23.44 (21.29,25.79)	23.76 (21.79,25.95)	0.483	23.78 (21.80,25.95)	23.44 (21.31,25.77)	0.826
Recurrence history, *n*(%)				0.243			0.200			0.244
No	391 (74.48)	85 (80.19)	302 (73.12)		150 (78.53)	237 (72.26)		190 (77.55)	197 (71.90)	
Yes	134 (25.52)	21 (19.81)	111 (26.88)		41 (21.47)	91 (27.74)		55 (22.45)	77 (28.10)	
RETURBT, *n*(%)				0.644			0.949			0.975
No	356 (67.81)	76 (71.70)	276 (66.83)		131 (68.59)	221 (67.38)		167 (68.16)	185 (67.52)	
Yes	169 (32.19)	30 (28.30)	137 (33.17)		60 (31.41)	107 (32.62)		78 (31.84)	89 (32.48)	
Hematuresis, *n*(%)				**0.006**			0.055			**0.027**
No	62 (11.81)	3 (2.83)	58 (14.04)		14 (7.33)	47 (14.33)		19 (7.76)	42 (15.33)	
Yes	463 (88.19)	103 (97.17)	355 (85.96)		177 (92.67)	281 (85.67)		226 (92.24)	232 (84.67)	
Pedunculated tumor, *n*(%)				0.854			0.730			0.816
No	458 (87.24)	94 (88.68)	359 (86.92)		164 (85.86)	289 (88.11)		216 (88.16)	237 (86.50)	
Yes	67 (12.76)	12 (11.32)	54 (13.08)		27 (14.14)	39 (11.89)		29 (11.84)	37 (13.50)	
T stage, *n*(%)				0.945			0.058			0.515
Ta	199 (37.90)	43 (40.57)	154 (37.29)		85 (44.50)	112 (34.15)		101 (41.22)	96 (35.04)	
T1	269 (51.24)	52 (49.06)	213 (51.57)		82 (42.93)	183 (55.79)		116 (47.35)	149 (54.38)	
Tx^a^	57 (10.86)	11 (10.38)	46 (11.14)		24 (12.57)	33 (10.06)		28 (11.43)	29 (10.58)	
Sex, *n*(%)				0.441			0.253			0.081
Male	432 (82.29)	89 (83.96)	339 (82.08)		162 (84.82)	266 (81.10)		210 (85.71)	218 (79.56)	
Female	93 (17.71)	17 (16.04)	74 (17.92)		29 (15.18)	62 (18.90)		35 (14.29)	56 (20.44)	
Smoking, *n*(%)				**<0.001**			**<0.001**			**<0.001**
No	239 (45.52)	67 (63.21)	167 (40.44)		112 (58.64)	122 (37.20)		143 (58.37)	91 (33.21)	
Yes	286 (54.48)	39 (36.79)	246 (59.56)		79 (41.36)	206 (62.80)		102 (41.63)	183 (66.79)	
CCI, *n*(%)				**<0.001**			0.051			**<0.001**
Mild (0–1)	42 (8.00)	4 (3.77)	37 (8.96)		13 (6.81)	28 (8.54)		14 (5.71)	27 (9.85)	
Moderate (2–4)	357 (68.00)	57 (53.77)	296 (71.67)		119 (62.30)	234 (71.34)		152 (62.04)	201 (73.36)	
Severe (>4)	126 (24.00)	45 (42.45)	80 (19.37)		59 (30.89)	66 (20.12)		79 (32.24)	46 (16.79)	
Severe postirrigation reactions^b^, *n*(%)				0.277			0.081			**0.024**
No	372 (70.86)	69 (65.09)	299 (72.40)		125 (65.45)	243 (74.09)		160 (65.31)	208 (75.91)	
Yes	153 (29.14)	37 (34.91)	114 (27.60)		66 (34.55)	85 (25.91)		85 (34.69)	66 (24.09)	
Diameter exceeds 3 cm, *n*(%)				**0.020**			0.951			0.884
No	336 (64.00)	56 (52.83)	276 (66.83)		121 (63.35)	211 (64.33)		154 (62.86)	178 (64.96)	
Yes	189 (36.00)	50 (47.17)	137 (33.17)		70 (36.65)	117 (35.67)		91 (37.14)	96 (35.04)	
Tumor focality, *n*(%)				0.943			0.516			0.112
Unifocal	200 (38.10)	39 (36.79)	159 (38.50)		67 (35.08)	131 (39.94)		82 (33.47)	116 (42.34)	
Multifocal	325 (61.90)	67 (63.21)	254 (61.50)		124 (64.92)	197 (60.06)		163 (66.53)	158 (57.66)	
CIS, *n*(%)				0.803			0.765			0.703
No	490 (93.33)	99 (93.40)	385 (93.22)		179 (93.72)	305 (92.99)		227 (92.65)	257 (93.80)	
Yes	35 (6.67)	7 (6.60)	28 (6.78)		12 (6.28)	23 (7.01)		18 (7.35)	17 (6.20)	
WHO grade, *n*(%)				0.089			**<0.001**			**0.002**
Low grade	197 (37.52)	32 (30.19)	161 (38.98)		51 (26.70)	142 (43.29)		74 (30.20)	119 (43.43)	
High grade	328 (62.48)	74 (69.81)	252 (61.02)		140 (73.30)	186 (56.71)		171 (69.80)	155 (56.57)	
Tumor location, *n*(%)				0.643			0.767			0.082
Neck triangular region	57 (10.98)	11 (10.38)	46 (11.14)		21 (10.99)	36 (10.98)		26 (10.61)	31 (11.31)	
Diverticulum	8 (1.52)	2 (1.89)	6 (1.45)		3 (1.57)	5 (1.52)		6 (2.45)	2 (0.73)	
Periureteral area	106 (20.19)	17 (16.04)	86 (20.82)		35 (18.32)	68 (20.73)		41 (16.73)	62 (22.63)	
Broadly distributed	118 (22.48)	29 (27.36)	88 (21.31)		49 (25.65)	68 (20.73)		65 (26.53)	52 (18.98)	
Other regions	236 (44.95)	47 (44.34)	187 (45.28)		83 (43.46)	151 (46.04)		107 (43.67)	127 (46.35)	
Pathology, *n*(%)				0.488			0.221			0.124
Urothelial	479 (91.24)	96 (90.57)	378 (91.53)		170 (89.01)	304 (92.68)		217 (88.57)	257 (93.80)	
Squamous	11 (2.10)	3 (2.83)	7 (1.69)		5 (2.62)	5 (1.52)		7 (2.86)	3 (1.09)	
Glandular	28 (5.33)	6 (5.66)	22 (5.33)		14 (7.33)	14 (4.27)		18 (7.35)	10 (3.65)	
Nesting	2 (0.38)	0 (0.00)	2 (0.48)		0 (0.00)	2 (0.61)		1 (0.41)	1 (0.36)	
Sarcoma	1 (0.19)	0 (0.00)	1 (0.24)		0 (0.00)	1 (0.30)		0 (0.00)	1 (0.36)	
Micropapillary	1 (0.19)	0 (0.00)	1 (0.24)		1 (0.52)	0 (0.00)		1 (0.41)	0 (0.00)	
Plasmacytoid	1 (0.19)	0 (0.00)	1 (0.24)		0 (0.00)	1 (0.30)		0 (0.00)	1 (0.36)	
Neuroendocrine	2 (0.38)	1 (0.94)	1 (0.24)		1 (0.52)	1 (0.30)		1 (0.41)	1 (0.36)	

NMIBC, non-muscle-invasive bladder cancer; NRITR, nutritional risk index for tumor recurrence; NRIBF, nutritional risk index for BCG-treatment failure; NRITP, nutritional risk index for tumor progression; BMI, body mass index; CCI score, Charlson Comorbidity Index score; CIS, carcinoma in situ; ReTURBT, repeat transurethral resection of bladder tumor; PNI, the prognostic nutritional index; GNRI, geriatric nutritional risk index; NPS, the Naples prognostic score. ^a^The staging of the primary tumor is undetermined. ^b^Severe postirrigation reactions include tuberculosis, fever, myalgia, arthralgia, hematuria, and irritable lower urinary tract symptoms. Bold numbers indicate *p* < 0.05.

The Youden index from ROC analysis determined optimal nutritional index cut-offs for BCG failure, categorizing patients into high and low risk groups: NRITR at ≤−3.034 and >−3.034, NRIBF at ≤−2.43 and >−2.43, NRITP at ≤−3.08 and >−3.08, PNI at ≤54.70 and >54.70, GNRI at ≤101.45 and >101.45, and NPS at ≤2 and >2.

### 3.2 The correlations between tumor prognosis and the EAU2021

**Figure 1:**
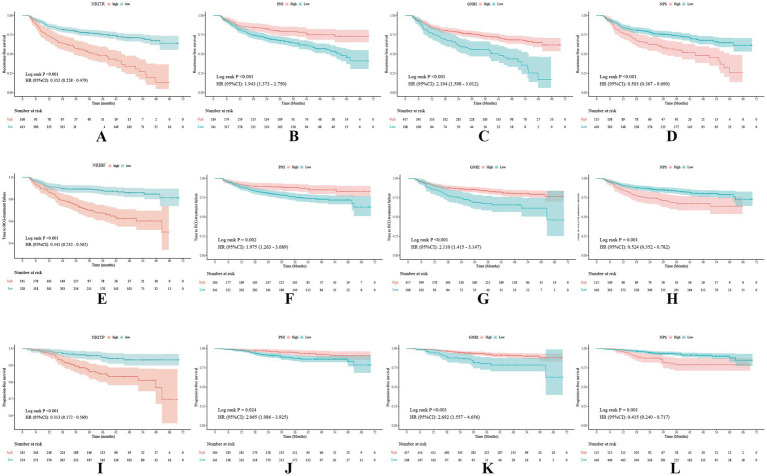
Kaplan–Meier survival curves of the NRITR **(A)**, NRIBF **(E)**, NRITP **(I)**, PNI **(B,F,J)**, GNRI **(C,G,K)**, and NPS **(D,H,L)** for tumor recurrence, BCG failure, and tumor progression. NRITR, nutritional risk index for tumor recurrence; NRIBF, nutritional risk index for BCG-treatment failure; NRITP, nutritional risk index for tumor progression; PNI, the prognostic nutritional index; GNRI, geriatric nutritional risk index; NPS, the Naples prognostic score.

During follow-up, elevated EAU2021 risk stratification in NMIBC correlated with higher recurrence, progression and poor BCG response (Supplementary Table 1). Kaplan–Meier analysis showed significant effects on RFS, TTF, and PFS (Supplementary Figure 4, Log rank *p* < 0.001). However, the overlapping Kaplan–Meier curves between intermediate and high-risk groups in predicting tumor progression indicated limited discriminatory ability. Univariable Cox regression showed EAU risk stratification significantly related to RFS and TTF, but not PFS (Figure 1).

### 3.3 The correlations between tumor recurrence and NRITR, PNI, GNRI, and NPS

**Figure 2:**
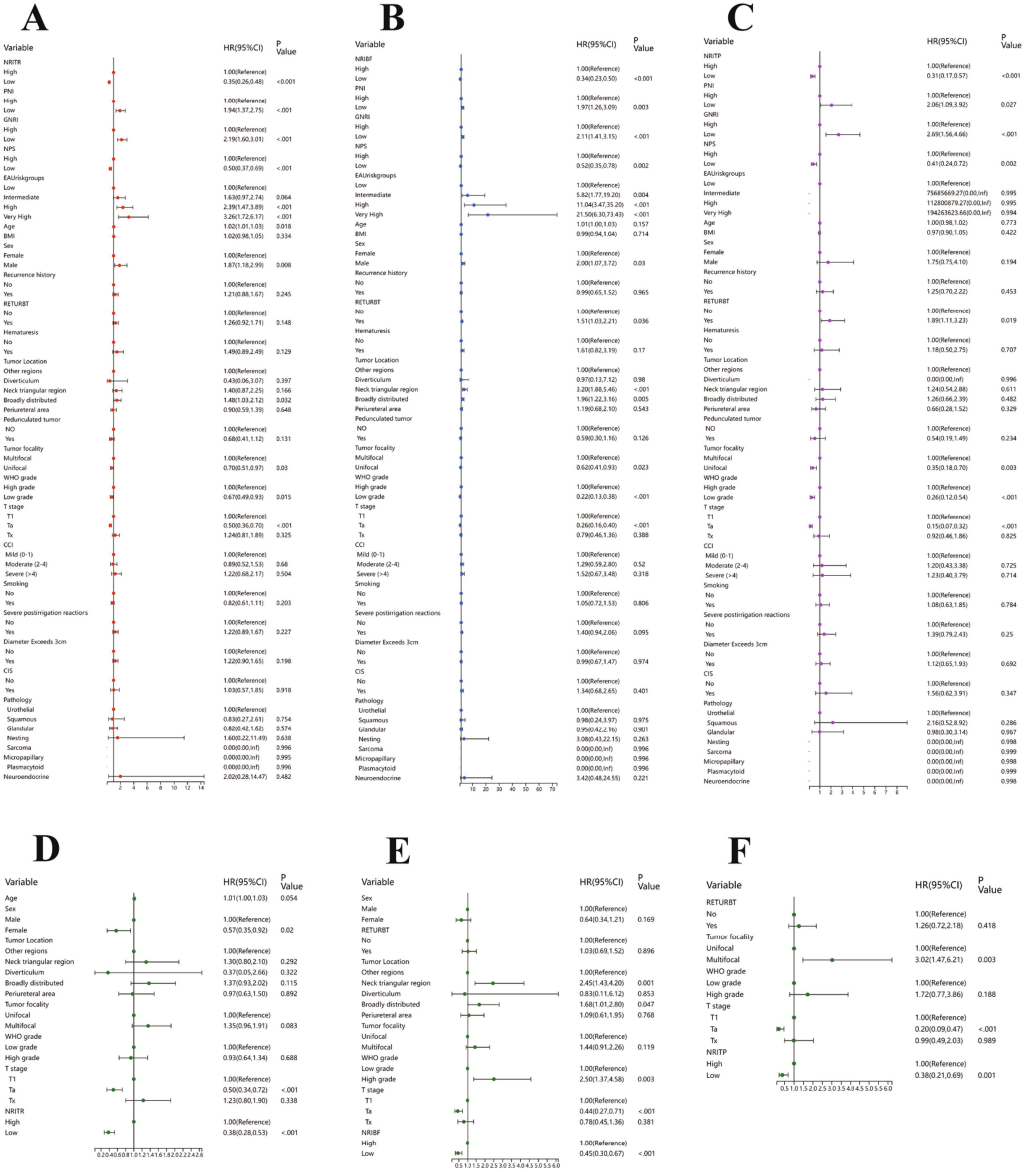
The results of the univariable regression models for RFS **(A)**, TTF **(B)** and PFS **(C)**, and the results of the multivariable Cox regression models based on NRITR **(D)**, NRIBF **(E)**, NRITP **(F)**. RFS, Recurrence-free survival; TTF, Time to BCG failure; PFS, Progression-free survival; NRITR, nutritional risk index for tumor recurrence; NRIBF, nutritional risk index for BCG-treatment failure; NRITP, nutritional risk index for tumor progression.

Kruskal-Wallis and Chi-square analyses linked higher NRITR (*p* < 0.001) and NPS (*p* < 0.001) with significantly higher recurrence rates, while lower PNI (*p* < 0.001) and GNRI (*p* < 0.001) showed the opposite (Table 1; Supplementary Table 2). Kaplan–Meier and log-rank tests confirmed significantly higher RFS for higher PNI, GNRI, and lower NRITR and NPS (Figure 2). Univariable and multivariable Cox regression analysis revealed NRITR (*p* < 0.001, HR = 0.38, 95%CI = 0.28–0.53), GNRI (*p* < 0.001, HR = 1.93, 95%CI = 1.38–2.70), NPS (*p* = 0.001, HR = 0.58, 95%CI = 0.42–0.80) and PNI (*p* = 0.009, HR = 1.62, 95%CI = 1.13–2.33) as independent predictors (Figure 1; Supplementary Figure 5).

### 3.4 The correlations between time to BCG-treatment failure and NRIBF, PNI, GNRI, and NPS

Differential analysis showed NRIBF (*p* < 0.001) and NPS (*p* = 0.009) were negatively linked to BCG response rates, while PNI (*p* = 0.005) and GNRI (*p* = 0.021) was positively correlated (Table 1; Supplementary Table 2). Kaplan–Meier and log-rank tests indicated significantly longer TTF for higher PNI and GNRI, and lower NRIBF and NPS (Figure 1). In univariable Cox regression analysis, NRIBF (*p* < 0.001), PNI (*p* = 0.003), GNRI (*p* < 0.001) and NPS (*p* = 0.002) were significantly associated with TTF. In multivariable analysis, NRIBF (*p* < 0.001, HR = 0.45, 95%CI = 0.30–0.67), GNRI (*p* = 0.008, HR = 1.76, 95%CI = 1.16–2.68) and NPS (*p* = 0.007, HR = 0.56, 95%CI = 0.37–0.85) independently predict TTF (Figure 2; Supplementary Figure 5).

### 3.5 The correlations between progression-free survival and NRITP, PNI, GNRI and NPS

Differential analysis indicated that lower NRITP (*p* < 0.001) and NPS (*p* = 0.019), and higher PNI (*p* = 0.020) and GNRI (*p* = 0.024) correlated with reduced progression rates (Table 1; Supplementary Table 2). Kaplan–Meier curves and the log-rank test, demonstrated a notable increase in PFS for patients with higher PNI, GNRI and lower NRITP and NPS (Figure 1). Univariable Cox regression analysis further confirmed the significant association of NRITP (*p* < 0.001), PNI (*p* = 0.027), GNRI (*p* < 0.001) and NPS (*p* = 0.002) with PFS. Multivariable analysis indicated that NRITP (*p* = 0.001, HR = 0.38, 95%CI = 0.21–0.69), GNRI (*p* = 0.007, HR = 2.15, 95%CI = 1.24–3.75) and NPS (*p* = 0.009, HR = 0.47, 95%CI = 0.27–0.83) could independently predict tumor progression (Figure 2; Supplementary Figure 5).

### 3.6 Validation of the accuracy and reliability of the nutritional indicators and EAU2021 model

**Figure 3:**
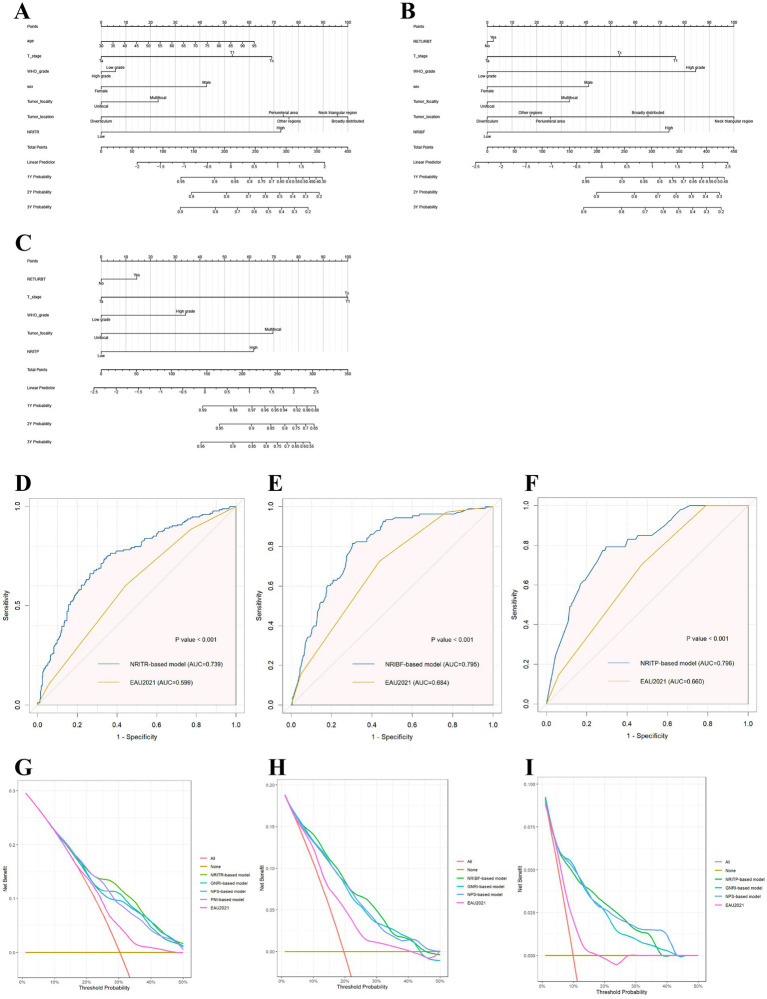
The nomograms based on NRIs according to the results of Cox regression models in predicting RFS **(A)**, TTF **(B)**, and PFS **(C)**, respectively; and the ROC plots of the NRIs-based models and the EAU2021 model in RFS **(D)**, TTF **(E)**, and PFS **(F)**; and the decision curve analysis on the net benefit from the nutritional models and the EAU2021 model in predicting RFS **(G)**, TTF **(H)**, and PFS **(I)**. RFS, Recurrence-free survival; TTF, Time to BCG failure; PFS, Progression-free survival; NRI, nutritional risk index.

The multivariable Cox regression analysis highlighted the roles of NRIs, PNI, GNRI, and NPS for predicting tumor prognosis. Therefore, the prognostic nomograms were developed accordingly (Figure 3).

As showed in Table 2, the area under the curve (AUC) for the EAU2021 model for predicting RFS, TTF, and PFS were 0.599, 0.684, and 0.660, respectively. With nutritional biomarkers added, the AUC of EAU2021 model increased largely (Figure 3). Notably, the NRI-based models demonstrated the highest predictive power for RFS (AUC = 0.739), TTF (AUC = 0.795), and PFS (AUC = 0.796). The C-index confirmed the models’ precision in predicting RFS (C-index = 0.693), TTF (C-index = 0.767), and PFS (C-index = 0.788). Decision Curve analysis further revealed the more net benefit the NRI-based models would bring than EAU2021 or other models for RFS and TTF. When consider tumor progression, the NRITP-based model and NPS-based model would bring similar net benefit, more than other models (Figure 3).

**Table 2:** The AUC values and C-indexes of nutritional risk models and EAU2021 model, in predicting RFS, TTF and PFS, respectively.

Models	RFS		TTF		PFS	
	AUC	C-index	AUC	C-index	AUC	C-index
The NRITP-based model					0.796 (0.738,0.855)	0.788
The NRIBF-based model			0.795 (0.751,0.839)	0.767		
The NRITR-based model	0.739 (0.693,0.785)	0.693				
The GNRI-based model	0.711 (0.664,0.758)	0.674	0.779 (0.735,0.824)	0.757	0.792 (0.730,0.854)	0.789
The NPS-based model	0.700 (0.653,0.747)	0.669	0.775 (0.729,0.822)	0.755	0.785 (0.721,0.849)	0.784
The PNI-based model	0.698 (0.651,0.745)	0.665				
EAU2021 + NRIBF			0.740 (0.689,0.790)	0.710		
EAU2021 + NRITP					0.716 (0.653,0.780)	0.702
EAU2021 + NRITR	0.674 (0.625,0.722)	0.638				
EAU2021 + GNRI	0.643 (0.594,0.691)	0.618	0.699 (0.650,0.748)	0.681	0.702 (0.637,0.766)	0.692
EAU2021 + NPS	0.639 (0.590,0.689)	0.614	0.704 (0.653,0.754)	0.689	0.691 (0.622,0.760)	0.685
EAU2021 + PNI	0.632 (0.585,0.680)	0.608				
EAU2021	0.599 (0.551,0.646)	0.592	0.684 (0.637,0.732)	0.669	0.660 (0.599,0.721)	0.647
NRIBF			0.648 (0.596,0.699)	0.623		
NRITP					0.636 (0.571,0.702)	0.630
NRITR	0.623 (0.583,0.663)	0.587				
GNRI	0.589 (0.550,0.628)	0.561	0.579 (0.531,0.627)	0.568	0.602 (0.534,0.670)	0.602
PNI	0.584 (0.543,0.625)	0.560				
NPS	0.583 (0.543,0.623)	0.558	0.584 (0.544,0.624)	0.559	0.595 (0.527,0.663)	0.602

RFS, recurrence-free survival; TTF, time to BCG-treatment failure; PFS, progression-free survival; EAU, European Association of Urology; NRITR, nutritional risk index for tumor recurrence; NRIBF, nutritional risk index for BCG-treatment failure; NRITP, nutritional risk index for tumor progression; GNRI, geriatric nutritional risk index; NPS, the Naples prognostic score; PNI, prognostic nutritional index; AUC, the area under the curve.

## 4 Discussion

Nutrition profoundly influences the prognosis of cancer patients, yet the prognostic significance of nutritional indicators is often overlooked in clinical practice, particularly in the early stages of cancer. It has been suggested that malnutrition is associated with a dampening of both innate and adaptive immune responses (13). Studies have demonstrated that BCa patients with preoperative malnutrition or nutritional risk exhibit poorer postoperative quality of life, higher complication rates, and significantly lower survival rates compared to well-nourished patients (8, 14). BCG regulates T cell responses for tumor killing, with circulating immune cells supporting its efficacy (5, 15). Malnutrition may impede the effectiveness of BCG immunotherapy by fostering an immunosuppressive environment (16). This study reveals that patients with lower nutritional risk indicators exhibited significantly prolonged RFS, TTF and PFS (*p* < 0.05), underscoring the critical role of nutritional assessment in guiding RC or bladder-preservation strategies. The EAU2021 model showed significant differences in tumor outcomes, confirming our dataset’s reliability. Notably, using Lasso-Cox regression, we identified the prognostic relevance of blood-based indexes, including albumin, total protein, glutamyl transpeptidase, cholesterol, GGT, and RDW-CV, in predicting BCG response for NMIBC. The novel contribution of this study lies in constructing the NRIs and demonstrating their independent prognostic value for BCG responsiveness. Furthermore, we systematically validated that established nutritional indicators (GNRI, NPS and PNI) provide robust predictive power for clinical outcomes in NMIBC. Importantly, our nutritional risk indicator-based prognostic models significantly outperformed the EAU 2021 risk stratification model in predictive accuracy, offering actionable evidence to optimize personalized treatment strategies and clinical decision-making.

The body’s nutritional status regulates key metabolic pathways (including protein, glucose, and lipid metabolism), inflammatory responses, and immune function, which collectively influence therapeutic sensitivity and prognosis in cancer patients (17). Notably, studies have demonstrated that BCa-driven metabolic reprogramming promotes tumor proliferation and confers drug resistance through these pathways (18–20). Serum albumin is one of the most widely used indicators for assessing malnutrition risk. Decrease in albumin levels, not only reflect inadequate liver synthesis but also indicate compromised immune function and limited antitumor responses, potentially contributing to poor prognosis in malignancies (21). Previous studies of BCa had shown that low preoperative serum albumin levels could independently predict increased postoperative complications and poor prognosis (22, 23). A meta-analysis of 18 BCa studies demonstrated that preoperative low albumin was significantly associated with inferior overall survival (OS) (HR = 1.88), cancer-specific survival (CSS) (HR = 1.69), 30-day postoperative complications (HR = 1.55), and 90-day mortality (HR = 2.87) (24). Composed of albumin and globulins, TP is also a parameter used to assess patients’ nutritional status. During cancer progression, increased metabolism and cachexia may lead to reduced availability of amino acids and limited protein synthesis, resulting in decreased levels of TP in blood (25). Enkobahry et al. (26) demonstrated significant correlations between malnutrition and serum albumin/total protein/hemoglobin levels, though TP showed weaker associations compared to total protein and hemoglobin. Notably, our prior study revealed that low total protein independently predicted worse RFS in NMIBC (HR = 0.62, 95%CI:0.41–0.95) (27). GGT, a stable serum hepatic enzyme, regulates glutathione and cysteine metabolic homeostasis through the *γ*-glutamyl cycle and serves as a key biomarker for oxidative stress surveillance, with demonstrated positive correlation to metabolic syndrome (28). Studies have shown GGT levels have prognostic value in malignancies, including BCa. Georgios et al. (29) conducted a retrospective analysis of 324 RC-treated BCa patients, revealing that elevated GGT correlated with poorer ECOG performance status, aggressive tumor pathology (high-grade/stage), elevated preoperative CRP/hepatic enzymes, and served as an independent predictor of all-cause mortality (*p* = 0.043). RDW serves as an indicator of anemia. During tumor progression, elevated cytokines (e.g., interleukin-6) and oxidative stress impair erythropoiesis, increase erythrocyte fragility, induce functional iron deficiency, and disrupt energy metabolism, thereby promoting the development of anemia (30). Approximately 40% of cancer patients exhibit pre-existing cancer-related anemia before antitumor treatment (31). In BCa, elevated RDW has been identified as an independent risk factor for BCG instillation recurrence in NMIBC patients (HR = 2.0, 95%CI 1.01–3.98, *p* = 0.047), with significant associations observed between high RDW and increased mortality, reduced hemoglobin, elevated C-reactive protein, decreased red blood cell counts, and advanced T-stage (32, 33). As for cholesterol, hypocholesterolemia could affect the fluidity of cell membranes, reduce the mobility of cell surface receptors and their ability to transmit transmembrane signals, making it difficult for immunocompetent cells to destroy cancer cells through membrane changes (34). Studies have found that high cholesterol intake is positively correlated with the risk of BCa development and poor prognosis (35). Elevated serum cholesterol levels show a significant association with shortened CCS in BCa patients (36). Integrating these evidences, the screening results of the Lasso-Cox regression in this study are supported by robust theoretical foundations.

Considering both albumin levels and body weight change, GNRI is a robustly validated tool for quantitative assessing nutrition-related prognosis risk (37). While studies suggested serum albumin may reflect acute disease and inflammation more prominently, the combination with body weight change enhancing the GNRI as a comprehensive reflection of nutritional-related prognostic risks in oncology patients, particularly at an advanced age (37). Preliminary evidence supports combining MNA and GNRI in nutritional screening, with the simpler GNRI outperforming MNA in prognostic value for nutritional status, patient outcomes, and complication risks (5, 37). Studies has pointed the potential prognostic role of GNRI regarding BCa. A recent retrospective study on 292 individuals has identified GNRI as an independent predictor for RFS (HR = 2.108, *p* = 0.004) and PFS (HR = 2.155, *p* = 0.019) in elderly NMIBC patients (12). PNI and NPS are also recognized as promising nutritional indicators, and have been validated across various cancers (38, 39). PNI has been extensively reported in BCa literature, where previous retrospective studies had identified low preoperative PNI levels as an independent risk factor of poorer RFS, OS and CSS in NMIBC patients (11, 40). NPS, a system evaluating prognosis with factors like albumin, cholesterol, and inflammatory cell ratio, was developed from a colorectal cancer study (41). In a retrospective study of upper tract urothelial carcinoma patients post-radical nephroureterectomy, NPS predicted OS, CSS, and PFS independently, but its role in BCa is unknown (10). Currently, we observed that GNRI, PNI, NPS could predict the prognosis of NMIBC patients treated with BCG effectively. Especially, GNRI, NPS and PNI were found independently correlated with RFS, while GNRI and NPS was also independent factor of TTF and PFS.

Based on Cox survival analysis, we developed a prognostic model using nutritional risk indicators incorporating clinicopathological factors (*p* < 0.05 in univariate regression) to predict RFS, TTF, and PFS in NMIBC patients. These models were further validated against the EAU2021 model in our patient cohort. In the results of univariable analysis, age, history of reTURBT, tumor location, tumor focality, T stage and WHO grade, exhibited statistical prognostic value, which was consistent with previous findings (2). Our results revealed that incorporating nutritional indicators significantly improves the predictive performance of the EAU2021 model. Among these nutritional risk models, the NRITR, NRIBF, and NRITP models achieved the highest AUC values for predicting RFS, TTF, and PFS, respectively, highlighting their accuracy. The NRITR (C-index = 0.692) and NRIBF (C-index = 0.767) models showed optimal discriminative ability, while the NRITP model had slightly lower PFS predictive power (C-index = 0.788) than the GNRI-based model (C-index = 0.789). For RFS and TTF prediction, decision curve analysis revealed superior clinical net benefit for NRITR and NRIBF over the EAU2021 and other nutritional risk models. For PFS prediction, NTITP and NPS models showed comparable clinical benefit, outperforming other models. Notably, the Lasso-derived nutritional risk index integrated only four peripheral blood markers, offering simpler composition and easier calculation than the EAU2021 model, enhancing clinical practicality.

We recognize several limitations inherent in our study. First, as a single-center retrospective study, the generalizability of our findings may be limited by institution-specific patient characteristics and treatment heterogeneity. Second, only preoperative data were collected, limiting the reflection of patients’ changing nutritional and immune statuses over the disease course. Third, the availability of only the 2004/2016 WHO grading system from our institution prevented validation of the EORTC model. Therefore, the findings of this study require further validation with larger sample sizes, diverse populations, and optimized modeling approaches to enhance their clinical utility. Future multicenter prospective studies incorporating more comprehensive clinical and pathological variables are recommended to improve the model’s predictive performance and clinical applicability.

## 5 Conclusion

The Lasso-Cox regression may offer superior value in selecting prognostic biomarkers for NMIBC. The Lasso-Cox regression-based NRIs enhance prognostic stratification for BCG-treated NMIBC, outperforming existing blood-based nutritional risk indicators and the EAU2021 model. Incorporation of blood nutritional indicators into clinical practice could optimization of personalized NMIBC treatment strategies and clinical decision-making. Further validation is warranted.

## Data Availability

The raw data supporting the conclusions of this article will be made available by the authors, without undue reservation.

## References

[1] Bray F Laversanne M Sung H Ferlay J Siegel RL Soerjomataram I. Global cancer statistics 2022: Globocan estimates of incidence and mortality worldwide for 36 cancers in 185 countries. CA Cancer J Clin. (2024) 74:229–63. doi: 10.3322/caac.21834 38572751

[2] Lenis AT Lec PM Chamie K Mshs MD. Bladder Cancer: a review. JAMA. (2020) 324:1980. doi: 10.1001/jama.2020.17598, PMID: 33201207

[3] Babjuk M Burger M Capoun O Cohen D Compérat EM Dominguez Escrig JL. European Association of Urology guidelines on non–muscle-invasive bladder Cancer (ta, T1, and carcinoma in situ). Eur Urol. (2022) 81:75–94. doi: 10.1016/j.eururo.2021.08.010, PMID: 34511303

[4] Witjes JA. Management of Bcg Failures in superficial bladder Cancer: a review. Eur Urol. (2006) 49:790–7. doi: 10.1016/j.eururo.2006.01.017 16464532

[5] Van Puffelen JH Keating ST Oosterwijk E van der Heijden AG Netea MG Joosten LAB. Trained immunity as a molecular mechanism for Bcg immunotherapy in bladder cancer. Nat Rev Urol. (2020) 17:513–25. doi: 10.1038/s41585-020-0346-4, PMID: 32678343

[6] Wang H Ding W Jiang G Gou Y Sun C Chen Z. Eortc risk tables are more suitable for Chinese patients with nonmuscle-invasive bladder cancer than Aua risk stratification. Medicine. (2018) 97:e12006. doi: 10.1097/Md.0000000000012006, PMID: 30200080 PMC6133586

[7] Planas M Álvarez-Hernández J León-Sanz M Celaya-Pérez S Araujo K García de Lorenzo A. Prevalence of hospital malnutrition in cancer patients: a sub-analysis of the Predyces® study. Support Care Cancer. (2016) 24:429–35. doi: 10.1007/s00520-015-2813-7, PMID: 26099900

[8] Tobert CM Hamilton-Reeves JM Norian LA Hung C Brooks NA Holzbeierlein JM. Emerging impact of malnutrition on surgical patients: literature review and potential implications for cystectomy in bladder Cancer. J Urol. (2017) 198:511–9. doi: 10.1016/j.juro.2017.01.087, PMID: 28286066 PMC5705177

[9] Madden AM Smith S. Body composition and morphological assessment of nutritional status in adults: a review of anthropometric variables. J Hum Nutr Diet. (2016) 29:7–25. doi: 10.1111/jhn.12278, PMID: 25420774

[10] Ye J Chen Z Pan Y Liao X Wang X Zhang C. The prognostic value of preoperative Naples prognostic score in upper tract urothelial carcinoma patients after radical Nephroureterectomy. Nutr Cancer. (2024) 76:80–8. doi: 10.1080/01635581.2023.2279218, PMID: 37941300

[11] Cui J Chen S Bo Q Wang S Zhang N Yu M. Preoperative prognostic nutritional index and nomogram predicting recurrence-free survival in patients with primary non-muscle-invasive bladder cancer without carcinoma in situ. Onco Targets Ther. (2017) 10:5541–50. doi: 10.2147/Ott.S146990, PMID: 29200869 PMC5702160

[12] Wu J Cheng X Yang H Xiao S Xu L Zhang C. Geriatric nutritional risk index as a prognostic factor in elderly patients with non-muscle-invasive bladder cancer: a propensity score-matched study. Int Urol Nephrol. (2024) 56:1627–37. doi: 10.1007/s11255-023-03905-6, PMID: 38177927

[13] Marik PE Zaloga GP. Immunonutrition in high-risk surgical patients. J Parenter Enter Nutr. (2010) 34:378–86. doi: 10.1177/0148607110362692, PMID: 20631383

[14] Munbauhal G Drouin SJ Mozer P Colin P Phé V Cussenot O. Malnourishment in bladder cancer and the role of immunonutrition at the time of cystectomy: an overview for urologists. BJU Int. (2014) 114:177–84. doi: 10.1111/bju.12529, PMID: 24410904

[15] Han J Gu X Li Y Wu Q. Mechanisms of Bcg in the treatment of bladder cancer-current understanding and the prospect. Biomed Pharmacother. (2020) 129:110393. doi: 10.1016/j.biopha.2020.110393, PMID: 32559616

[16] Schaible UE Kaufmann SHE. Malnutrition and infection: complex mechanisms and global impacts. PLoS Med. (2007) 4:e115. doi: 10.1371/journal.pmed.0040115, PMID: 17472433 PMC1858706

[17] Zitvogel L Pietrocola F Kroemer G. Nutrition, inflammation and cancer. Nat Immunol. (2017) 18:843–50. doi: 10.1038/ni.3754, PMID: 28722707

[18] Whyard T Waltzer WC Waltzer D Romanov V. Metabolic alterations in bladder cancer: applications for cancer imaging. Exp Cell Res. (2016) 341:77–83. doi: 10.1016/j.yexcr.2016.01.005, PMID: 26808412

[19] Deng J Peng M Zhou S Xiao D Hu X Xu S. Metformin targets Clusterin to control lipogenesis and inhibit the growth of bladder cancer cells through Srebp-1c/Fasn axis. Signal Transduct Target Ther. (2021) 6:98. doi: 10.1038/s41392-021-00493-8, PMID: 33649289 PMC7921554

[20] Wang L Xu T Yang X Liang Z Zhang J Li D. Immunosuppression induced by glutamine deprivation occurs via activating Pd-L1 transcription in bladder Cancer. Front Mol Biosci. (2021) 8:687305. doi: 10.3389/fmolb.2021.687305, PMID: 34805266 PMC8602840

[21] Moujaess E Fakhoury M Assi T Elias H el Karak F Ghosn M. The therapeutic use of human albumin in cancer patients’ management. Crit Rev Oncol Hematol. (2017) 120:203–9. doi: 10.1016/j.critrevonc.2017.11.008, PMID: 29198333

[22] Djaladat H Bruins HM Miranda G Cai J Skinner EC Daneshmand S. The association of preoperative serum albumin level and American Society of Anesthesiologists (Asa) score on early complications and survival of patients undergoing radical cystectomy for urothelial bladder cancer. BJU Int. (2014) 113:887–93. doi: 10.1111/bju.12240, PMID: 23906037

[23] Bhalla RG Wang L Chang SS Tyson MD. Association between preoperative albumin levels and length of stay after radical cystectomy. J Urol. (2017) 198:1039–45. doi: 10.1016/j.juro.2017.05.066, PMID: 28533006

[24] Liu J Wang F Li S Huang W Jia Y Wei C. The prognostic significance of preoperative serum albumin in urothelial carcinoma: a systematic review and meta-analysis. Biosci Rep. (2018) 38:Bsr20180214. doi: 10.1042/Bsr20180214, PMID: 29685957 PMC6435544

[25] Zhang Z Pereira SL Luo M Matheson EM. Evaluation of blood biomarkers associated with risk of malnutrition in older adults: a systematic review and Meta-analysis. Nutrients. (2017) 9:829. doi: 10.3390/nu9080829, PMID: 28771192 PMC5579622

[26] Enkobahry A Sime T Kene K Mateos T Dilnesa S Zawdie B. Blood biomarkers as potential malnutrition screening alternatives among adult patients with cancer on treatment in oncology unit of Jimma tertiary hospital: a cross-sectional analysis. Bmc. Nutrition. (2023) 9:694. doi: 10.1186/s40795-023-00694-0, PMID: 36869395 PMC9982783

[27] Ye J Tang C Wu R Tang Y Yin H Bai Y. Preoperative blood-based nutritional biomarkers as significant prognostic factors after intravesical Bcg therapy in patients with non-muscle-invasive bladder cancer. World J Urol. (2024) 42:428. doi: 10.1007/s00345-024-05148-1, PMID: 39037600

[28] Raya-Cano E Molina-Luque R Vaquero-Abellán M Molina-Recio G Jiménez-Mérida R Romero-Saldaña M. Metabolic syndrome and transaminases: systematic review and meta-analysis. Diabetol Metab Syndr. (2023) 15:220. doi: 10.1186/s13098-023-01200-z, PMID: 37899468 PMC10614379

[29] Gakis G Schmid MA Hassan F Stenzl A Renninger M. The predictive and prognostic value of Precystectomy serum gamma-Glutamyltransferase levels in patients with invasive bladder Cancer. Clin Genitourin Cancer. (2022) 20:e310–6. doi: 10.1016/j.clgc.2022.02.006 35314137

[30] Means RT Krantz SB. Progress in understanding the pathogenesis of the anemia of chronic disease[J]. Blood. (1992) 80:1639–47. doi: 10.1182/blood.V80.7.1639.1639, PMID: 1391934

[31] Ludwig H Van Belle S Barrett-Lee P Birgegård G Bokemeyer C Gascón P. The European Cancer Anaemia survey (Ecas): a large, multinational, prospective survey defining the prevalence, incidence, and treatment of anaemia in cancer patients. Eur J Cancer. (2004) 40:2293–306. doi: 10.1016/j.ejca.2004.06.019 15454256

[32] Ma W Mao S Bao M Wu Y Guo Y Liu J. Prognostic significance of red cell distribution width in bladder cancer. Transl Androl Urol. (2020) 9:295–302. doi: 10.21037/tau.2020.03.08, PMID: 32420135 PMC7215002

[33] Fukuokaya W Kimura T Miki J Kimura S Watanabe H Bo F. Red cell distribution width predicts time to recurrence in patients with primary non-muscle-invasive bladder cancer and improves the accuracy of the Eortc scoring system. Urol Oncol. (2020) 38:638.e15–23. doi: 10.1016/j.urolonc.2020.01.016, PMID: 32184059

[34] Oliver MF. Serum cholesterol--the knave of hearts and the joker. Lancet. (1981) 2:1090–5. doi: 10.1016/s0140-6736(81)91286-1 6118533

[35] Yang S Ye Z Ning J Wang P Zhou X Li W. Cholesterol metabolism and urinary system tumors. Biomedicines. (2024) 12:1832. doi: 10.3390/biomedicines12081832, PMID: 39200296 PMC11351655

[36] Ferro M Lucarelli G De Cobelli O Dolce P Terracciano D Musi G. A risk-group classification model in patients with bladder cancer under neoadjuvant cisplatin-based combination chemotherapy. Future Oncology (London, England). (2021) 17:3987–94. doi: 10.2217/fon-2020-1298, PMID: 34278815

[37] Cereda E Pedrolli C. The geriatric nutritional risk index. Curr Opin Clin Nutr Metabolic Care. (2009) 12:1–7. doi: 10.1097/Mco.0b013e3283186f59, PMID: 19057180

[38] Wu H Fu M Xie X Yang J Liu Y du F. Naples prognostic score, a novel prognostic score for patients with high- and intermediate-risk gastrointestinal stromal tumours after surgical resection. World J Surg Oncol. (2022) 20:63. doi: 10.1186/s12957-022-02526-0, PMID: 35232450 PMC8886834

[39] Sun K Chen S Xu J Li G He Y. The prognostic significance of the prognostic nutritional index in cancer: a systematic review and meta-analysis. J Cancer Res Clin Oncol. (2014) 140:1537–49. doi: 10.1007/s00432-014-1714-3, PMID: 24878931 PMC11823704

[40] Bi H Shang Z Jia C Wu J Cui B Wang Q. Predictive values of preoperative prognostic nutritional index and systemic immune-inflammation index for long-term survival in high-risk non-muscle-invasive bladder Cancer patients: a single-Centre retrospective study. Cancer Manag Res. (2020) 12:9471–83. doi: 10.2147/Cmar.S259117, PMID: 33061634 PMC7534864

[41] Galizia G Lieto E Auricchio A Cardella F Mabilia A Podzemny V. Naples prognostic score, based on nutritional and inflammatory status, is an independent predictor of long-term outcome in patients undergoing surgery for colorectal Cancer. Dis Colon Rectum. (2017) 60:1273–84. doi: 10.1097/Dcr.0000000000000961, PMID: 29112563

