# Corrigendum: Prognostic impact of nutritional indicators based on Lasso-Cox regression for non-muscle-invasive bladder cancer

**DOI:** 10.3389/fnut.2025.1630197

**Published:** 2025-06-12

**Authors:** Junjiang Ye, Yandong Xie, Biao Ran, Ping Han

**Affiliations:** Department of Urology, Institute of Urology, West China Hospital, Sichuan University, Chengdu, China

**Keywords:** non-muscle-invasive bladder cancer, Lasso-Cox regression, geriatric nutritional risk index, prognostic nutritional index, Naples prognostic score

In the published article, there was an error in the Supplementary Materials, Supplementary Figure 2. When generating the ten-fold cross-validation plot for the Lasso regression, we employed the incorrect methodology, resulting in an error in the final visualization. The correct Supplementary Figure 2 caption and figure has been published in the original article.

In the published article, there was an error in the Supplementary Materials. The headings for the Supplementary Figures were incorrectly written as “1.1 Supplementary Figures 1”, “1.2 Supplementary Figures 2”, “1.3 Supplementary Figures 3”, “1.4 Supplementary Figures 4” and “1.5 Supplementary Figures 5”. The correct headings should have been written as “1.1 Supplementary Figure 1”, “1.2 Supplementary Figure 2”, “1.3 Supplementary Figure 3”, “1.4 Supplementary Figure 4” and “1.5 Supplementary Figure 5”.

The authors apologize for this error and state that this does not change the scientific conclusions of the article in any way. The original article has been updated.

