# Supplementary material for: Prognostic impact of nutritional indicators based on Lasso-Cox regression for non-muscle-invasive bladder cancer
Source: Front Nutr. 2025 Apr 28;12:1560655. doi: 10.3389/fnut.2025.1560655 (PMC12066686; doi:10.3389/fnut.2025.1560655)
Supplement: Supplementary file 1 [file Data_Sheet_1.docx]

Supplementary Materials

# Supplementary Figures

## Supplementary Figure 1


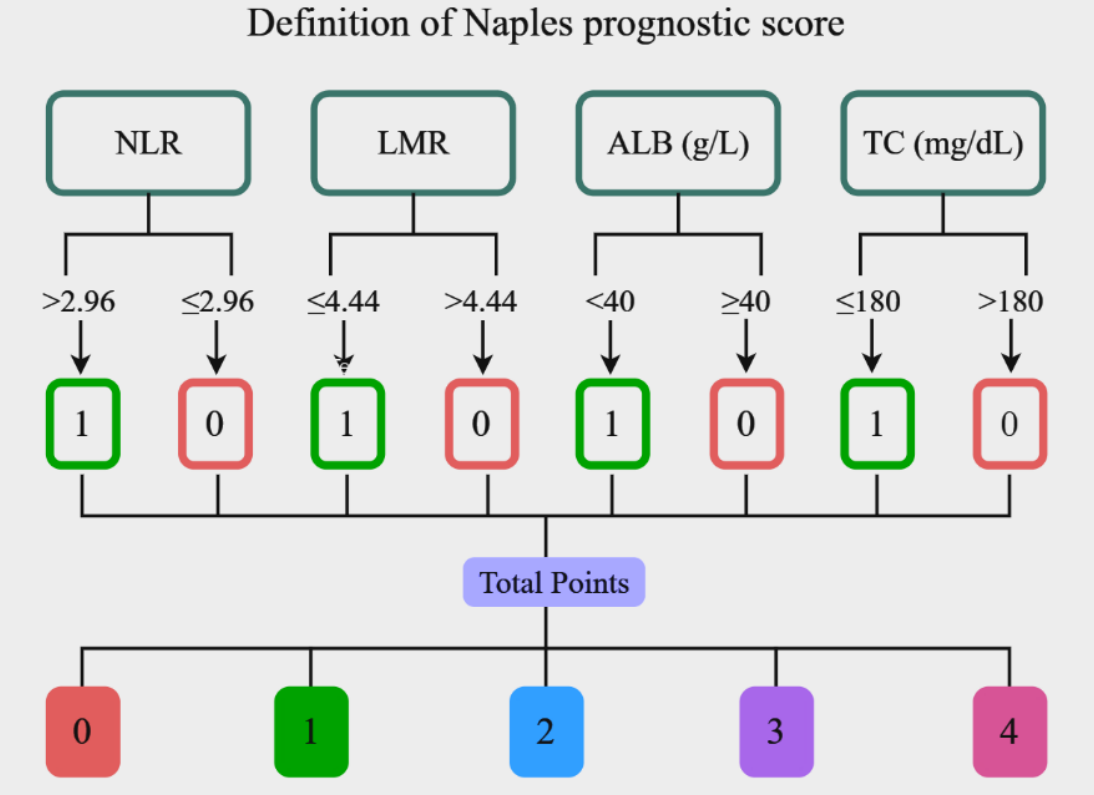


**Supplementary Figure 1.** Definition of Naples prognosis score.

## Supplementary Figure 2


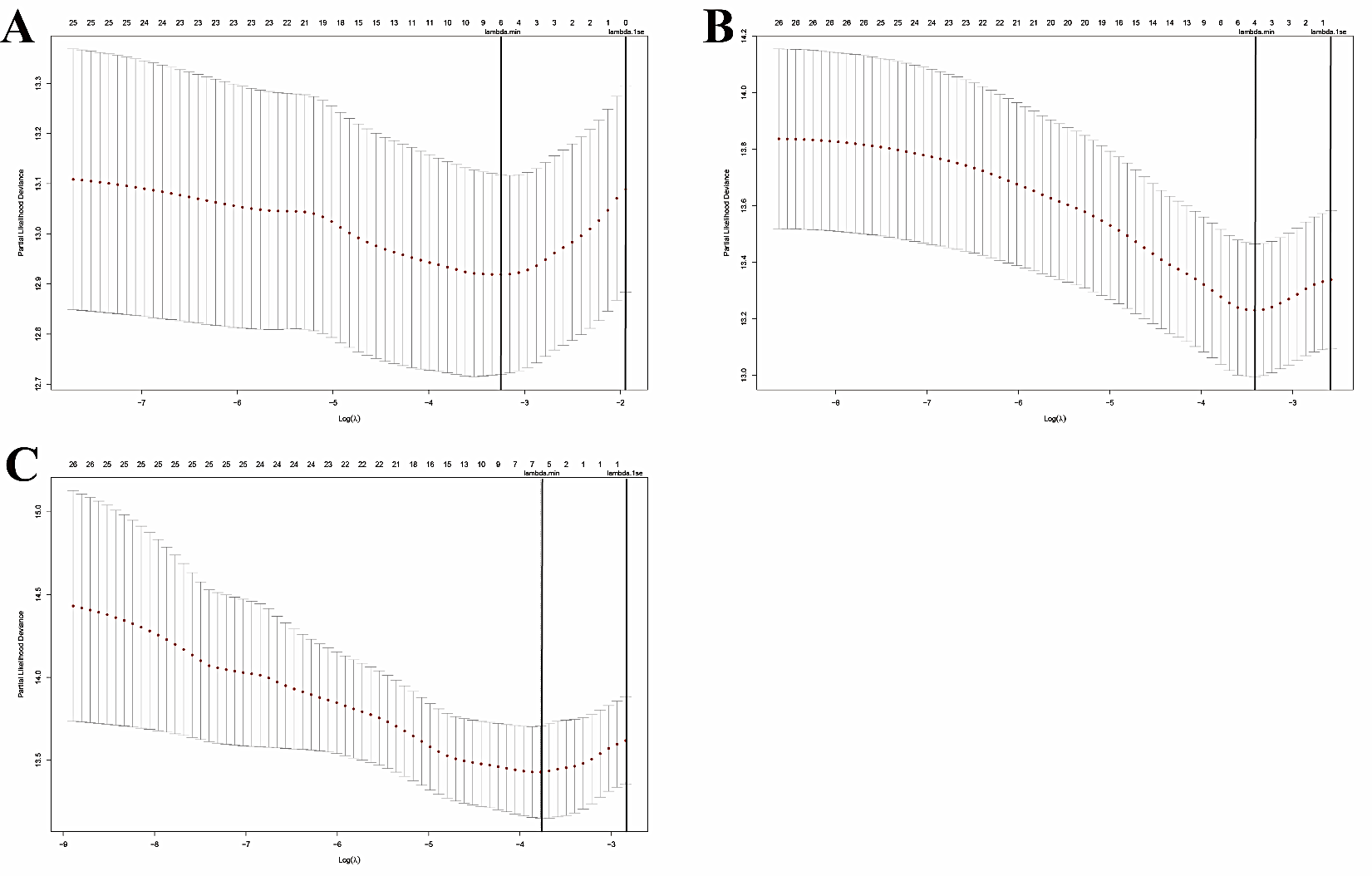
**Supplementary Figure 2.** Lasso-Cox Regression with 10-Fold Cross-Validation on RFS (A), TTF (B) and PFS (C). RFS: Recurrence-free survival; TTF: Time to BCG failure; PFS: Progression-free survival; lamda.min: Minimum Lambda; lamda.1se: Lambda within 1 Standard Error of the Minimum.

## Supplementary Figure 3


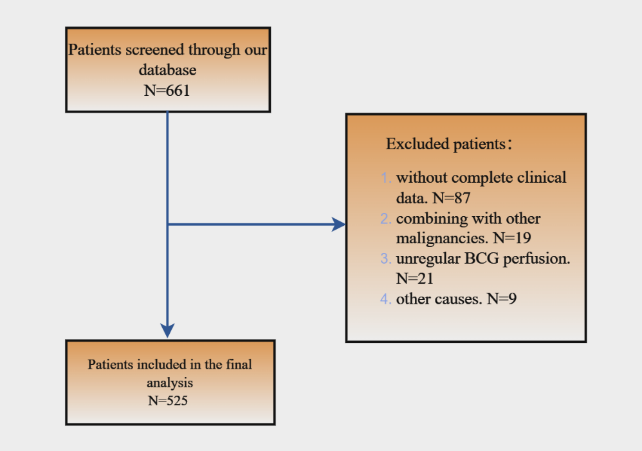


**Supplementary Figure 3.** Study flowchart: the number of patients included and excluded based on the inclusion and exclusion criteria in the present study.

## Supplementary Figure 4


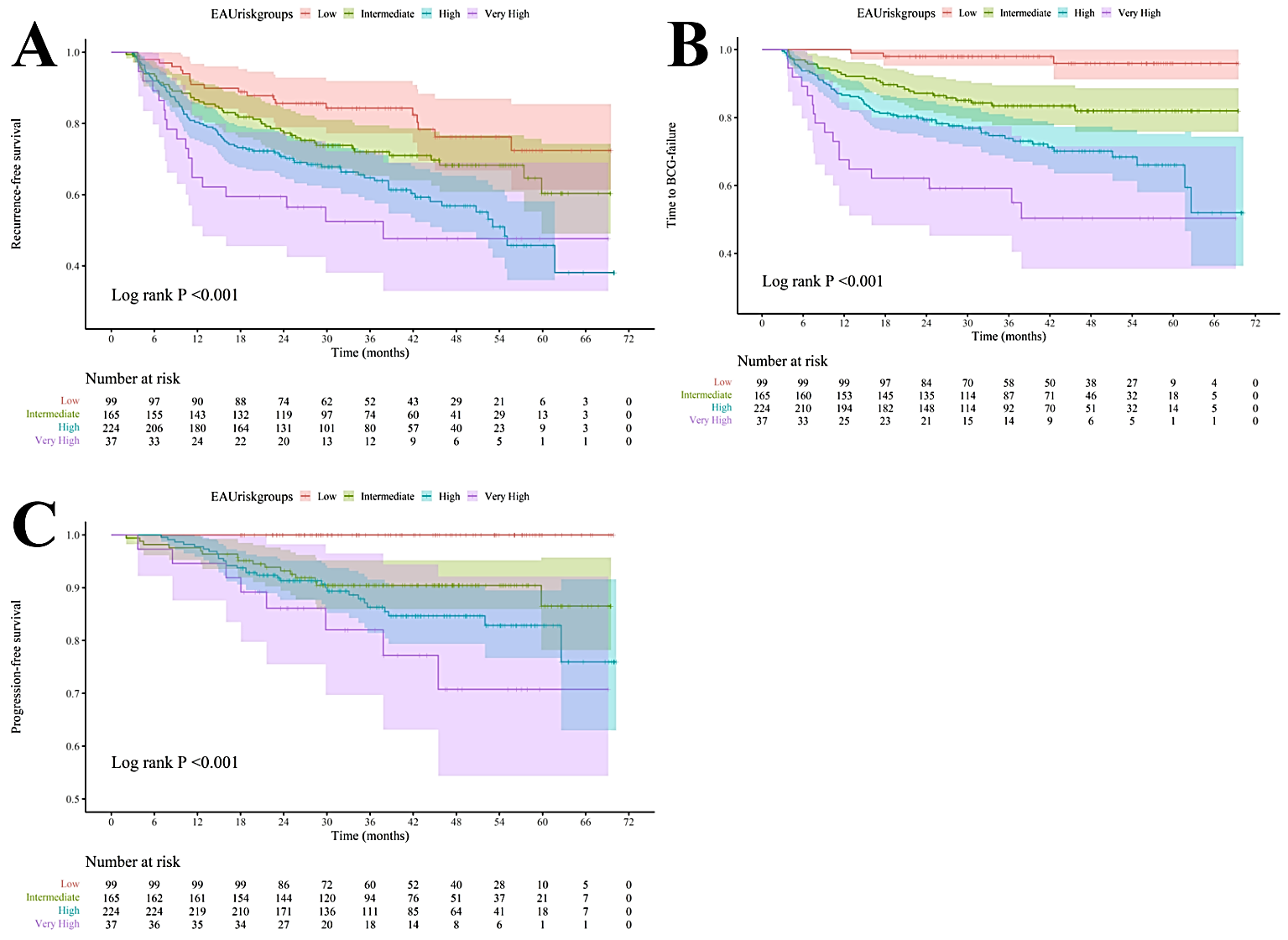
**Supplementary Figure 4.** Kaplan-Meier survival curves of the EAU2021 model (A: tumor recurrence, B: BCG-treatment failure, C: tumor progression).

##
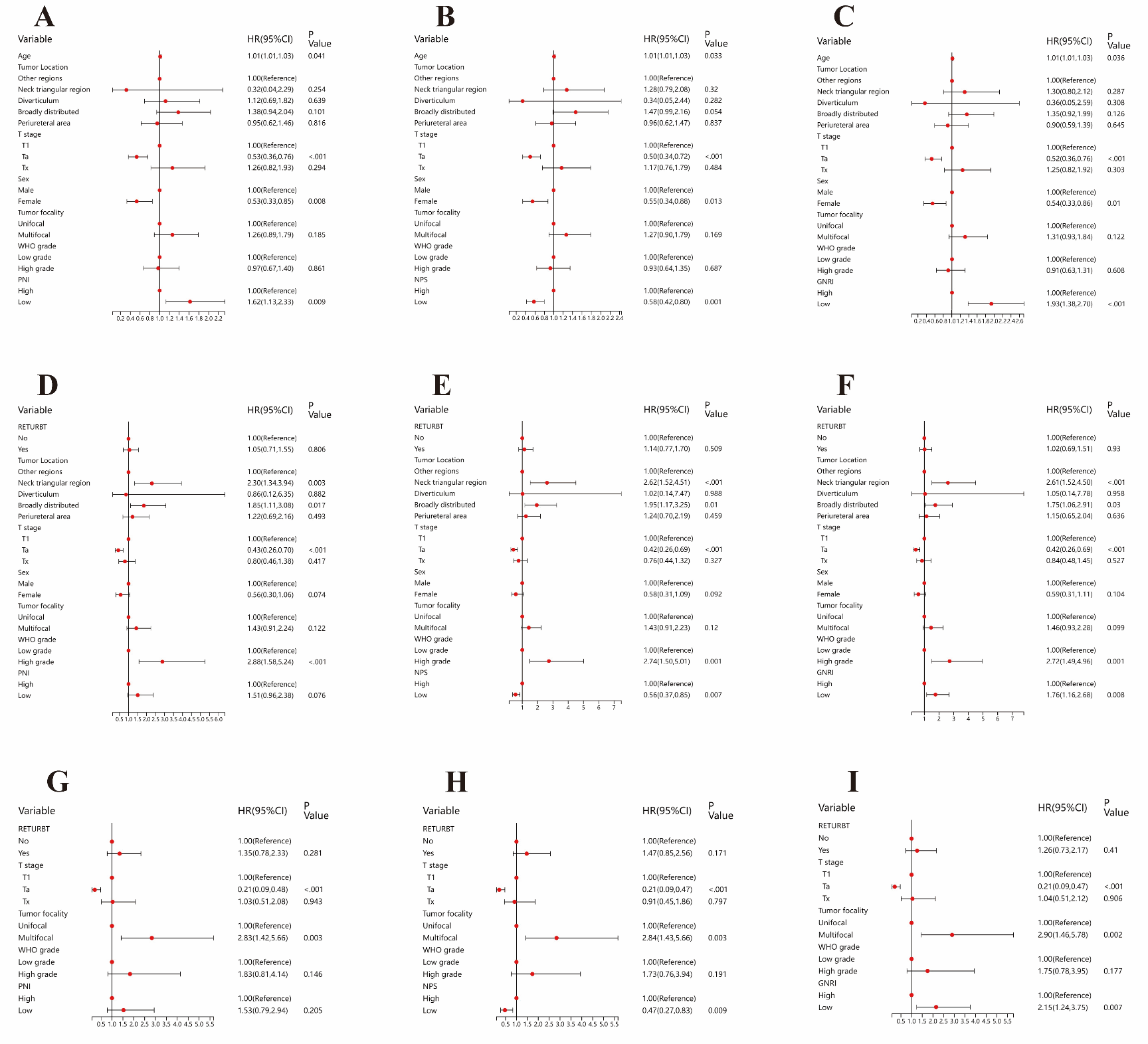
Supplementary Figure 5

**Supplementary Figure 5.** The results of the multivariable Cox regression models based on PNI (RFS: A, TTF: D, PFS: G), NPS (RFS: B, TTF: E, PFS: H), GNRI (RFS: C, TTF: F, PFS: I). RFS: Recurrence-free survival; TTF: Time to BCG failure; PFS: Progression-free survival; PNI: prognostic nutritional index; GNRI: geriatric nutritional risk index; NPS: the Naples prognostic score.

# Supplementary Tables

## Supplementary Table 1- Demographic and clinical data of patients with NMIBC stratified by EAU NMIBC risk groups.

| Characteristics | Total (n = 525) | Low (n = 99) | Intermediate (n = 165) | High (n = 224) | Very High (n = 37) | *P* |
| --- | --- | --- | --- | --- | --- | --- |
|  |  |  |  |  |  |  |
| Tumor recurrence, n(%) |  |  |  |  |  | **0.001** |
| No | 349 (66.48) | 79 (79.80) | 115 (69.70) | 136 (60.71) | 19 (51.35) |  |
| Yes | 176 (33.52) | 20 (20.20) | 50 (30.30) | 88 (39.29) | 18 (48.65) |  |
| Tumor progression, n(%) |  |  |  |  |  | **<.001** |
| No | 471 (89.71) | 99 (100.00) | 149 (90.30) | 194 (86.61) | 29 (78.38) |  |
| Yes | 54 (10.29) | 0 (0.00) | 16 (9.70) | 30 (13.39) | 8 (21.62) |  |
| BCG response |  |  |  |  |  | **<.001** |
| Responsive | 416(79.24) | 96 (96.97) | 138 (83.64) | 162 (72.32) | 20 (54.05) |  |
| Unresponsive | 25 (4.76) | 0 (0.00) | 7 (4.24) | 14 (6.25) | 4 (10.81) |  |
| Late relapsing | 84 (16.00) | 3 (3.03) | 20 (12.12) | 48 (21.43) | 13 (35.14) |  |
| PNI, M (Q₁, Q₃) | 52.65 (48.35, 56.45) | 54.95 (50.73,57.50) | 53.10 (48.35,56.80) | 51.27 (47.69,55.57) | 53.30 (48.50,57.15) | **<.001** |
| GNRI, M (Q₁, Q₃) | 109.53 (102.93, 115.57) | 113.49 (108.21,117.42) | 110.33 (103.51,115.85) | 107.22 (101.30,112.58) | 108.66 (100.41,116.97) | **<.001** |
| NPS, n(%) |  |  |  |  |  | **0.004** |
| 0 | 104 (19.81) | 27 (12.05) | 44 (26.67) | 27 (27.27) | 6 (16.22) |  |
| 1 | 147 (28.00) | 58 (25.89) | 43 (26.06) | 34 (34.34) | 12 (32.43) |  |
| 2 | 159 (30.29) | 76 (33.93) | 45 (27.27) | 26 (26.26) | 12 (32.43) |  |
| 3 | 81 (15.43) | 47 (20.98) | 23 (13.94) | 6 (6.06) | 5 (13.51) |  |
| 4 | 34 (6.48) | 16 (7.14) | 10 (6.06) | 6 (6.06) | 2 (5.41) |  |
| Age, M (Q₁, Q₃) | 67.00 (57.00, 74.00) | 61.00 (50.00,67.00) | 66.00 (57.00,73.00) | 69.00 (59.00,77.00) | 75.00 (65.00,79.00) | **<.001** |
| BMI, M (Q₁, Q₃) | 23.66 (21.63, 25.80) | 24.58 (22.46,26.29) | 23.83 (21.77,25.95) | 23.07 (21.26,25.44) | 23.63 (21.38,25.54) | 0.050 |
| Recurrence history, n(%) |  |  |  |  |  | **0.003** |
| No | 391 (74.48) | 62 (62.63) | 118 (71.52) | 181 (80.80) | 30 (81.08) |  |
| Yes | 134 (25.52) | 37 (37.37) | 47 (28.48) | 43 (19.20) | 7 (18.92) |  |
| ReTURBT, n(%) |  |  |  |  |  | **<.001** |
| No | 356 (67.81) | 86 (86.87) | 110 (66.67) | 145 (64.73) | 15 (40.54) |  |
| Yes | 169 (32.19) | 13 (13.13) | 55 (33.33) | 79 (35.27) | 22 (59.46) |  |
| Hematuresis, n(%) |  |  |  |  |  | 0.182 |
| No | 62 (11.81) | 18 (18.18) | 16 (9.70) | 24 (10.71) | 4 (10.81) |  |
| Yes | 463 (88.19) | 81 (81.82) | 149 (90.30) | 200 (89.29) | 33 (89.19) |  |
| Tumor location, n(%) |  |  |  |  |  | 0.291 |
| Neck triangular region | 57 (10.86) | 5 (5.05) | 23 (13.94) | 22 (9.82) | 7 (18.92) |  |
| Diverticulum | 8 (1.52) | 2 (2.02) | 2 (1.21) | 3 (1.34) | 1 (2.70) |  |
| Periureteral area | 106 (20.19) | 24 (24.24) | 33 (20.00) | 45 (20.09) | 4 (10.81) |  |
| Other regions | 236 (44.95) | 50 (50.51) | 70 (42.42) | 101 (45.09) | 15 (40.54) |  |
| Broadly distributed | 118 (22.48) | 18 (18.18) | 37 (22.42) | 53 (23.66) | 10 (27.03) |  |
| Pedunculated tumor, n(%) |  |  |  |  |  | **0.013** |
| No | 458 (87.24) | 80 (80.81) | 143 (86.67) | 206 (91.96) | 29 (78.38) |  |
| Yes | 67 (12.76) | 19 (19.19) | 22 (13.33) | 18 (8.04) | 8 (21.62) |  |
| T stage, n(%) |  |  |  |  |  | **<.001** |
| Ta | 269 (51.24) | 96 (96.97) | 105 (63.64) | 66 (29.46) | 2 (5.41) |  |
| T1 | 199 (37.90) | 1 (1.01) | 18 (10.91) | 147 (65.62) | 33 (89.19) |  |
| Tx^a^ | 57 (10.86) | 2 (2.02) | 42 (25.45) | 11 (4.91) | 2 (5.41) |  |
| Sex, n(%) |  |  |  |  |  | 0.880 |
| Male | 432 (82.29) | 80 (80.81) | 139 (84.24) | 183 (81.70) | 30 (81.08) |  |
| Female | 93 (17.71) | 19 (19.19) | 26 (15.76) | 41 (18.30) | 7 (18.92) |  |
| CCI, n(%) |  |  |  |  |  | **<.001** |
| Mild (0-1) | 42 (8.00) | 20 (20.20) | 12 (7.27) | 8 (3.57) | 2 (5.41) |  |
| Moderate (2-4) | 357 (68.00) | 67 (67.68) | 125 (75.76) | 147 (65.62) | 18 (48.65) |  |
| Severe (>4) | 126 (24.00) | 12 (12.12) | 28 (16.97) | 69 (30.80) | 17 (45.95) |  |
| Smoking, n(%) |  |  |  |  |  | 0.077 |
| No | 239 (45.52) | 35 (35.35) | 79 (47.88) | 111 (49.55) | 14 (37.84) |  |
| Yes | 286 (54.48) | 64 (64.65) | 86 (52.12) | 113 (50.45) | 23 (62.16) |  |
| Severe postirrigation reactions^b^, n(%) |  |  |  |  |  | 0.173 |
| No | 372 (70.86) | 78 (78.79) | 119 (72.12) | 150 (66.96) | 25 (67.57) |  |
| Yes | 153 (29.14) | 21 (21.21) | 46 (27.88) | 74 (33.04) | 12 (32.43) |  |
| Diameter Exceeds 3cm, n(%) |  |  |  |  |  | **<.001** |
| No | 336 (64.00) | 93 (93.94) | 109 (66.06) | 117 (52.23) | 17 (45.95) |  |
| Yes | 189 (36.00) | 6 (6.06) | 56 (33.94) | 107 (47.77) | 20 (54.05) |  |
| Tumor focality, n(%) |  |  |  |  |  | **0.002** |
| Unifocal | 200 (38.10) | 50 (50.51) | 70 (42.42) | 72 (32.14) | 8 (21.62) |  |
| Multifocal | 325 (61.90) | 49 (49.49) | 95 (57.58) | 152 (67.86) | 29 (78.38) |  |
| CIS, n(%) |  |  |  |  |  | **<.001** |
| No | 490 (93.33) | 99 (100.00) | 165 (100.00) | 205 (91.52) | 21 (56.76) |  |
| Yes | 35 (6.67) | 0 (0.00) | 0 (0.00) | 19 (8.48) | 16 (43.24) |  |
| WHO grade, n(%) |  |  |  |  |  | **<.001** |
| Low grade | 197 (37.52) | 99 (100.00) | 75 (45.45) | 22 (9.82) | 1 (2.70) |  |
| High grade | 328 (62.48) | 0 (0.00) | 90 (54.55) | 202 (90.18) | 36 (97.30) |  |
| Pathology, n(%) |  |  |  |  |  | **<.001** |
| Urothelial | 479 (91.24) | 98 (98.99) | 154 (93.33) | 200 (89.29) | 27 (72.97) |  |
| Squamous | 11 (2.10) | 0 (0.00) | 2 (1.21) | 9 (4.02) | 0 (0.00) |  |
| Glandular | 28 (5.33) | 1 (1.01) | 9 (5.45) | 15 (6.70) | 3 (8.11) |  |
| Nesting | 2 (0.38) | 0 (0.00) | 0 (0.00) | 0 (0.00) | 2 (5.41) |  |
| Sarcoma | 1 (0.19) | 0 (0.00) | 0 (0.00) | 0 (0.00) | 1 (2.70) |  |
| Micropapillary | 1 (0.19) | 0 (0.00) | 0 (0.00) | 0 (0.00) | 1 (2.70) |  |
| Plasmacytoid | 1 (0.19) | 0 (0.00) | 0 (0.00) | 0 (0.00) | 1 (2.70) |  |
| Neuroendocrine | 2 (0.38) | 0 (0.00) | 0 (0.00) | 0 (0.00) | 2 (5.41) |  |
| Abbreviations: NMIBC, non-muscle-invasive bladder cancer; BMI, body mass index; CCI score, Charlson Comorbidity Index score; CIS, carcinoma in situ; ReTURBT, repeat transurethral resection of bladder tumor.  Bold numbers indicate p < 0.05.  ^a^ The staging of the primary tumor is undetermined.  ^b^ Severe postirrigation reactions include tuberculosis, fever, myalgia, arthralgia, hematuria, and irritable lower urinary tract symptoms. | | | | | | |

## Supplementary Table 2 - PNI, GNRI and NPS of patients with NMIBC stratified by tumor recurrence, BCG failure and tumor progression.

|  | Total number | No tumor recurrence | Tumor recurrence | *P* | BCG response | BCG no response | Late relapse | *P* | No tumor progression | Tumor progression | *P* |
| --- | --- | --- | --- | --- | --- | --- | --- | --- | --- | --- | --- |
| Number | 525 | 349 | 176 |  | 416 | 25 | 84 |  | 471 | 54 |  |
| PNI, M (Q₁, Q₃) | 52.65(48.35,56.45) | 53.50 (49.55, 57.05) | 50.78 (45.43, 54.70) | **<.001** | 53.00(48.65,56.91) | 52.95(50.25,57.10) | 50.92(46.49,54.36) | **0.005** | 52.90(48.50,56.70) | 50.67(46.65,54.70) | **0.020** |
| ALI, M (Q₁, Q₃) | 498.96(352.78,711.02) | 513.58 (373.83, 724.15) | 465.36 (310.86, 656.27) | **0.010** | 502.22(357.20,716.39) | 562.05(346.15,810.10) | 469.42(336.57,611.85) | 0.187 | 508.54(360.88,716.55) | 422.49(294.41,603.86) | **0.014** |
| GNRI, M (Q₁, Q₃) | 109.53(102.93,115.57) | 110.40 (104.42, 116.06) | 107.33 (99.99, 114.77) | **<.001** | 110.19(103.88,115.76) | 108.58(104.09,115.31) | 105.82(100.16,114.93) | **0.021** | 109.97(103.41,115.68) | 106.39(99.74,114.03) | **0.024** |
| NPS, n(%) |  |  |  | **<.001** |  |  |  | **0.009** |  |  | **0.019** |
| 0 | 104(19.81) | 77 (22.06) | 27 (15.34) |  | 88(21.15) | 7(28.00) | 9(10.71) |  | 98(20.81) | 6(11.11) |  |
| 1 | 147(28.00) | 108 (30.95) | 39 (22.16) |  | 125(30.05) | 4(16.00) | 18(21.43) |  | 133(28.24) | 14(25.93) |  |
| 2 | 159(30.29) | 107 (30.66) | 52 (29.55) |  | 124(29.81) | 8(32.00) | 27(32.14) |  | 146(31.00) | 13(24.07) |  |
| 3 | 81(15.43) | 41 (11.75) | 40 (22.73) |  | 53(12.74) | 6(24.00) | 22(26.19) |  | 65(13.80) | 16(29.63) |  |
| 4 | 34(6.48) | 16 (4.58) | 18 (10.23) |  | 26(6.25) | 0(0.00) | 8(9.52) |  | 29(6.16) | 5(9.26) |  |
| Abbreviation：NMIBC, non-muscle-invasive bladder cancer; PNI, prognostic nutritional index; GNRI, geriatric nutritional risk index; NPS, the Naples prognostic score.  Bold numbers indicate p < 0.05. | | | | | | | | | | | |
